# Irritable Bowel Syndrome in the Elderly Population: A Comprehensive Review

**DOI:** 10.7759/cureus.68156

**Published:** 2024-08-29

**Authors:** Elva R Valtierra Oba, Ana C Anguiano Morán, Elizabeth Calderón Cortes, Myriam I Valtierra Oba, Barbara M Lemus Loeza, Alain Raimundo Rodríguez-Orozco

**Affiliations:** 1 Nursing, Universidad Michoacana de San Nicolás de Hidalgo, Morelia, MEX; 2 Geriatrics, Universidad Michoacana de San Nicolás de Hidalgo, Morelia, MEX; 3 Diabetes and Endocrinology, Universidad Michoacana de San Nicolás de Hidalgo, Morelia, MEX; 4 Allergy and Immunology, Universidad Michoacana de San Nicolás de Hidalgo, Morelia, MEX

**Keywords:** irritable bowel syndrome, probiotics, treatment of ibs, ibs subtypes, elderly population

## Abstract

Irritable bowel syndrome (IBS) is a fairly common functional digestive disorder; it occurs at any age but it is more common in adults and older adults. Patients experience a series of symptoms in which abdominal pain and changes in bowel movements stand out; some studies have revealed a possible association between IBS and psychological problems, such as anxiety and depression. Recent findings point to disorders of gut-brain interaction, disruption and alteration of gut microbiota and dysbiosis as key factors in the etiopathogenesis of IBS; aging is also one the factors involved. Most patients diagnosed with IBS required pharmacotherapy, greater caution needs to be considered when treating older patients because of the risk-benefit profile in the elderly. In this scenario, probiotics and non-pharmacological treatments appear as safe and accessible options. Clinicians must take into consideration the unique biopsychosocial factors in older adults when treating IBS. We aim to review critically recent literature on the topic of IBS as there is a need for consolidated guidelines.

## Introduction and background

According to the World Health Organization [1], every country in the world is experiencing growth in both the size and the proportion of older persons in their population; increased life expectancy and declining mortality levels are considered responsible for this phenomenon. The share of the global population aged 65 years or above is projected to rise from 10% in 2022 to 16% in 2050 [2]; this projection validates the need to understand aging better. Various factors and morphological and physiological modifications that act over time are responsible for the aging process of living beings, molecular and cellular damage affects biological organization decreasing physical and mental capacity, leading to illness and finally to death [1,3,4]. When an organism grows older, endogenous and exogenous damage accumulates, and as only some of the damage can be cleared or repaired, it leads to changes in functionally relevant biomolecules in ways that deleteriously affect cellular and organismal processes [4,5]. Geriatric syndromes are often present in older ages, they include frailty, urinary incontinence, falls, and pressure ulcers due to immobilization [1]. While gastrointestinal (GI) and digestive disorders can occur at any age, the prevalence of GI diseases increases with age due to physiological changes and the decline associated with aging. In this context, irritable bowel syndrome (IBS) emerges as a common functional GI disorder, affecting an estimated 10% to 20% of the elderly population [6].

IBS is a chronic affliction that presents itself often without an apparent structural or anatomical cause [7,8], its pathophysiology is not fully understood and seems to be multifactorial. Although these disorders can affect individuals regardless of age, gender, and race, women are four times as likely as men to suffer from IBS and it seems that there is a higher prevalence in the later stages of life, among the elderly [9]. Patients may experience an array of symptoms such as stomach pain, discomfort, flatulence, nausea, dyspepsia, reflux, and changing bowel patterns [7,10]. It has been proposed that IBS symptoms are related to certain alterations in the function and communications of the gut-brain axis that cause motility disturbance, visceral hypersensitivity, altered mucosal and immune function, upset gut microbiota, and altered central nervous system processing [11]. It is pertinent to note that the diagnosis of IBS is based on clinical symptoms and as such can be categorized based on the predominant bowel habit, and the treatment is generally based on subtype and symptom severity [11,12].

## Review

Survey methodology

**Figure 1:**
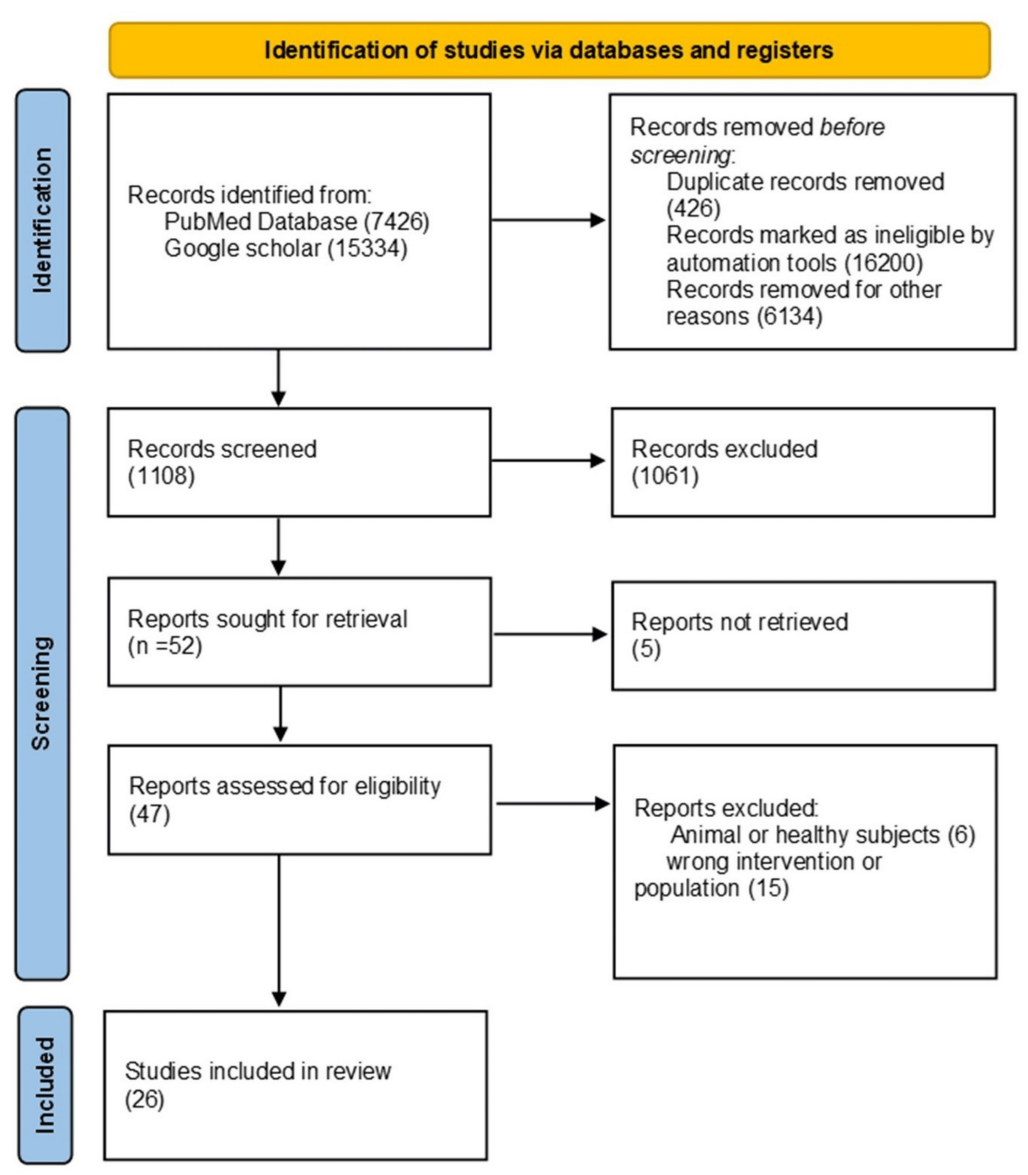
PRISMA chart for the search strategy PRISMA model from Page MJ, Mackensie JE, Bossuyt PM, Boutron I, Hoffmann TC, Mulrow CD, et al. The PRISMA 2020 statement: an updated guideline for reporting systematic reviews. BMJ. 2021, 29;372. doi: 101136/bmj.n71. PRISMA: Preferred Reporting Items for Systematic Reviews and Meta-Analyses

A literature review was conducted using the Google Scholar search engine and PubMed database. Relevant articles were identified using keywords and medical subject heading (MeSH) terms such as irritable bowel syndrome, elderly population, gastrointestinal tract disorders, management of bowel disorders, and gut microbiota. These keywords were combined with “AND”, “OR” and “NOT” Boolean operators in order to narrow down the results. In this review, the articles that were included have been published in the last five years that were related to human studies regardless of gender, race, or geography, prioritizing the elderly population. The selected papers were published in English. All types of studies were included, considering those that were available as free full-text articles. Articles that were not in English, had duplicate studies, were published before 2017, or the studies that included animals or healthy subjects were excluded from this review as shown in Figure 1.

Results

IBS is a collection of symptoms that are usually present simultaneously in a given period of time, including abdominal pain and fluctuations in bowel movements, such as bouts of diarrhea, constipation, or both. There are no discernable signs or evidence of damage in the digestive tract [13,14]. IBS is one of the most common functional GI disorders worldwide, these disorders are classified in a separate clinical domain to distinguish them from those of organic or motility nature. The Rome diagnostic criteria consider IBS to be a gut-brain interaction disarray that has a negative influence on quality of life (QoL) and social functioning [15,16]. Even though a specific GI disease limited to advanced age has not been identified as such, some ailments and disorders are more prevalent in this age group and may entail different approaches in consideration of the functions that are affected by aging, such as digestion, absorption, motility, hormone, and enzyme production [6,7]. Overall, few studies address IBS in the elderly population; information about patients with coexisting cardiovascular, neurologic, and other comorbidities common to this age group is limited. In older patients, systemic diseases, previous surgeries, medications, and their side effects can significantly alter the presentation of IBS patterns [4].

Prevalence

Several international reports point to IBS global prevalence within a range of 9% to 23% [14]; it is considered that the main difference between country prevalence rates relies primarily on diagnostic criteria rather than gender or age [16]. Prevalence rates by Rome IV criteria were lower than those using previous versions, it is also noteworthy that considering the increased lifespan and the rapidly aging population, the number of older adults with IBS is rising, with a prevalence of 9.7% and 7.5% in middle-aged and older adults, respectively [17]. According to the World Gastroenterology Organization [18], it is quite common to find classic IBS symptoms among the general population but most patients are not properly diagnosed, this can vouch for some of the variances in reported prevalence; for example, in México it was reported 16% of IBS prevalence by Rome II criteria which increased to 35% when community population was included in the appraisal; the available information suggests this is a similar occurrence in several countries as many reports and studies tend not to include community prevalence but only diagnosed patients.

Pathogenesis

**Figure 2:**
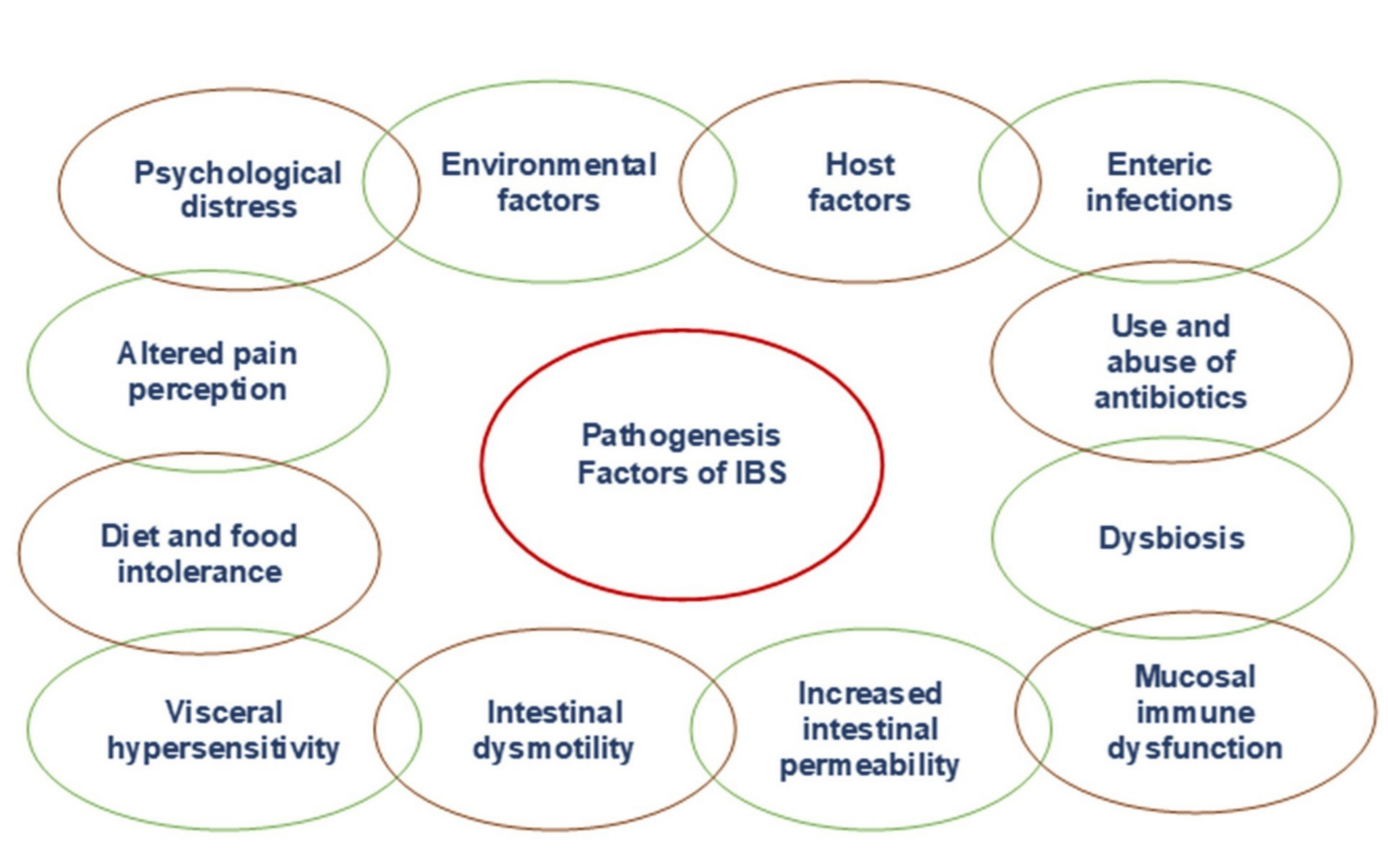
IBS contributing factors The figure displays the contributing factors associated with irritable bowel syndrome (IBS) pathogenesis (adapted from source [14]).

The etiology and pathophysiology of IBS are not completely elucidated; however, it is known that some factors can increase the risk of developing this condition. It has been noted that IBS pathogenesis is heterogeneous and traditionally related to either environmental or host factors such as infectious diseases or distress as shown in Figure 2.

It is reported in the literature that anxiety and depression are more common in IBS sufferers than in the healthy population [14]. Stress and psychological disorders can disrupt the brain-gut axis and increase corticotrophins which affects mood, digestive motility, and visceral sensitivity [8]. To this day, according to Ford and collaborators [16], it has not yet been established as a biomarker of disease thus IBS relies on a collection of self-reported symptoms. IBS is a heterogenous, chronic disease with a complex and multifactorial pathogenesis that is still not fully understood [19]. The Rome Foundation in its publications assumed a gradual shift from the classical definition of “Functional Gastrointestinal Disorders” to “Disorders of Gut-Brain Interaction,” highlighting the importance of pathophysiology processes such as dysbiosis, increased gut permeability, altered immune function, and neural and hormonal interaction between the brain and the gut [16,20]. The gut microbiota is regarded as a key player in the pathophysiology of IBS, and it is believed to be a consequence of the interplay between gut-brain axis disturbances, local immune changes, gut permeability, and inflammation in the gut wall [21]. There is evidence that suggests the microbiome has a role in the cause and pathogenesis of IBS [22] compared to healthy individuals, IBS patients have lower fecal microbial diversity, studies such as the trial conducted by Kim et al. [23] in South Korea demonstrated that gut bacterial dysbiosis is associated with IBS, but the causal relationship is yet to be determined.

Diagnosis

For IBS there is no test, in the majority of the cases, diagnosis is established on the basis of symptoms criteria and clinical history [16]. A complete and exhaustive clinical history is considered of great importance while assessing a patient, it is crucial to appraise more than the primary symptoms, to review various factors and other symptoms including extra-GI ones, and the general context for the symptoms that could have other explanation, it is also important to identify typical IBS features [18] as shown in Table 1. By not having a clear pathological or biochemical account and being associated with a variety of factors, IBS diagnosis is challenging [19]. IBS as a complex multifactorial entity requires a careful and wide approach regarding the diagnosis, it is elementary to exclude any organic disease and to take special consideration of comorbidities and polypharmacy in older persons [21].

**Table 1:** Features compatible with irritable bowel syndrome (IBS) The table shows patterns of abdominal pain and/or discomfort, with * marking those consistent with IBS (adapted from source [18]).

Pattern of abdominal pain or discomfort
Chronic duration*
Type of pain: intermittent* or continuous.
Previous pain episodes.*
Location of pain
Relief with defecation or passing of flatus.*
Other abdominal symptoms:
Bloating*
Distension*
Borborygmi
Flatulence
Nocturnal pain is unusual in IBS and is considered a warning sign.

As the diagnosis of IBS can not be confirmed by a specific test or a structural abnormality, it is usually made based on clinical symptom criteria [9]. The current symptom-based diagnostic criteria are the Rome IV criteria, which were developed by consensus among experts in functional GI disorders [20]. The term “abdominal discomfort” has been modified in the Rome criteria due to the imprecise nature of the term and because it has different meanings in different languages and among individuals; also the frequency of abdominal pain changed from three or more days per month to at least one day per week during the past three months [14,20]. It is paramount for the diagnosis that the patients describe their pain or discomfort in detail, including its time, intensity, and location. IBS patients may have any of four bowel patterns, which are classified under Rome IV [20] as shown in Table 2. While IBS symptoms are not significantly different across age groups, the elderly are more likely to have organic GI disease, hence, a careful diagnostic approach should be used in this particular group. It seems that clinicians are more likely to attribute GI symptoms in the elderly to an organic or medication-related etiology than to a functional disorder [7].

**Table 2:** The Rome Criteria for IBS and its subgroups IBS: irritable bowel syndrome

Rome IV criteria	
IBS with constipation	≥25% of bowel movements of Bristol Stool Form types 1 or 2, and <25% of Bristol Stool Form types 6 or 7
IBS with diarrhea	≥25% of bowel movements pf Bristol Stool Form types 6 or 7, and <25% of Bristol Stool Form types1 or 2
IBS with mixed stool pattern	≥25% of bowel movements of Bristol Stool Form types 1 or 2, and ≥25% of Bristol Stool Form types 6 or 7
IBS unclassified	Patients who meet the criteria for IBS, but do not fall into one of the other three subgroups according to the Bristol Stool Form type.

**Figure 3:**
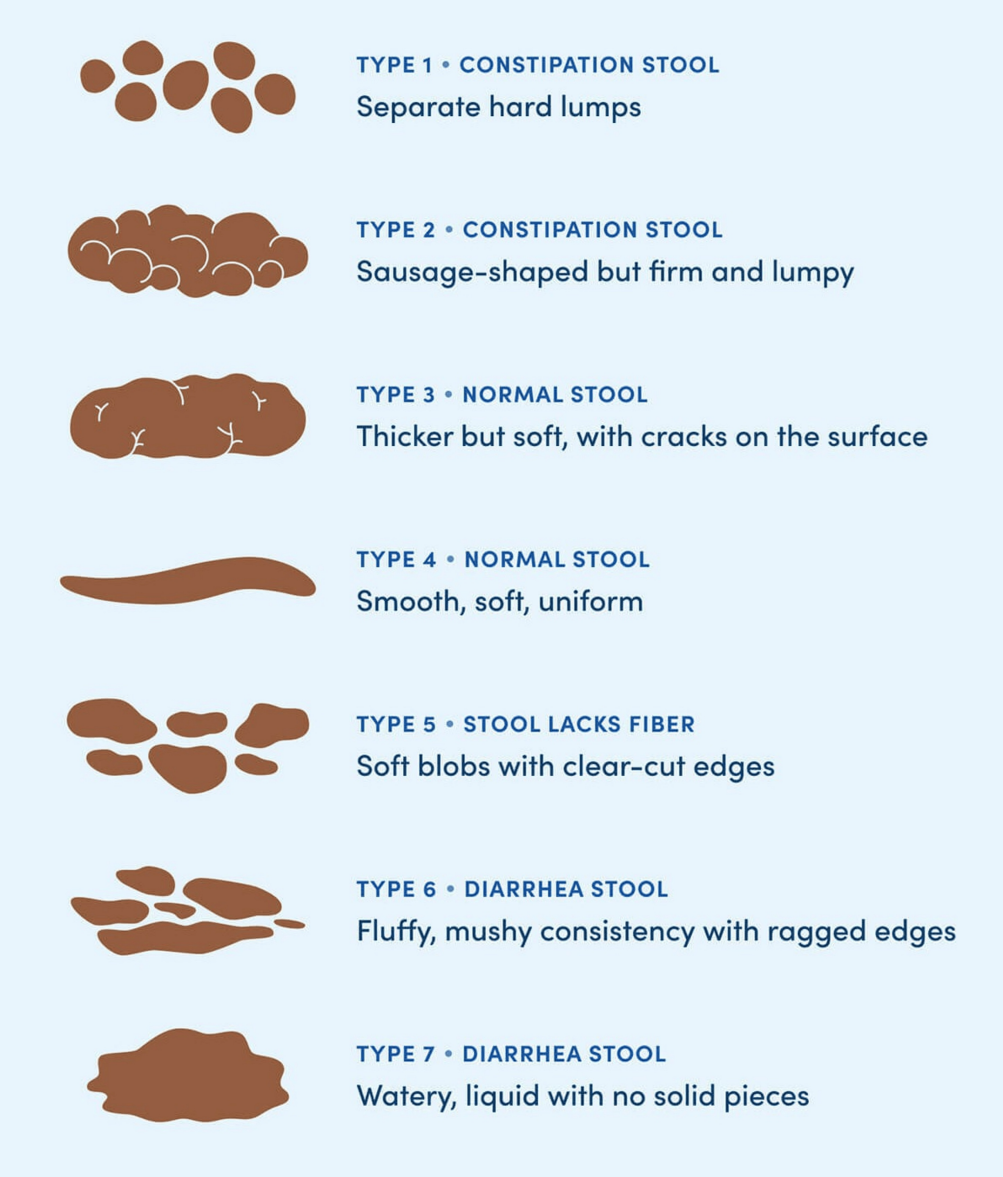
Bristol Stool Chart Assessment of fecal consistency scale from source [24].

Altered bowel habits constitute a criterion of IBS, with the Bristol Stool Form Scale (BSFS) as the recommended tool for the assessment of fecal consistency [24]. The type of stool or feces depends on the time it spends in the colon. The Bristol Stool Chart shows seven categories shown in Figure 3.

Recently it has been considered that a classification system based on a group of self-reported symptoms and stool types does not properly expose the complex nature of IBS [19,25]; thus the Rome Foundation developed the multidimensional clinical profile (MDCP), a framework that includes assessment of psychological factors and takes in consideration different clinical profile for each person in addition of the classical clinical symptoms and points out that the impact of IBS in QoL also varies within the individuals of a subgroup of classification, as a patient can start with one condition and six months later switches to another condition [25,26]. A clinical trial cohort proved that only one in four patients retained their baseline classification throughout the study evidencing considerable movement between subtypes of IBS [27]. This novel way of classification together with psychological profiles aims to optimize treatment and to generate better outcomes [25]. Studies have revealed a possible association between IBS and psychological problems, such as anxiety and depression. Mood disorders are much more common in IBS sufferers than among healthy people [28]. Furthermore, psychological distress in terms of depression and anxiety is a growing problem among older people, in this age group those disorders can lead to increased comorbidity with physical illness, considerable distress, and functional impairment [29].

Altered Gut Microbiota

The gut microbiota composition and function are stable in adulthood up to 65-70 years, and then the interindividual variability would be increased with the decline of biodiversity and a tendency to dysbiosis, leading to significant disturbance in host physiology [30]. The diversity of gut microbiota and the carriage of commensals such as *Bacteroides*, *bifidobacteria,* and *Lactobacillus* are found to be reduced while the levels of opportunists such as enterobacteria are increased in the elderly, these alterations may not necessarily be triggered by aging; it is speculated that the loss of microbiome diversity is rather correlated with aging-related frailty than with chronological age per se [31]. Disruptions to gut microbial composition, also known as gut dysbiosis, have been observed in several diseases, such as functional GI-related disorders and it is considered a focal determinant in IBS etiopathogenesis [32]. Severity IBS symptoms, both GI and psychological, have been associated with a distinct intestinal microbiota composition [22,33], microbial populations, such as *Lactobacillus, Bifidobacterium*, and *Faecalibacterium*, are significantly depleted, and pathogenic microbial populations such as *Clostridium difficile*, *Helicobacter pylori, and Escherichia coli* are often enriched in IBS patients [34]. Gut microbes interact with the host via several routes, including neural, neuroimmune, and neuroendocrine pathways [33], furthermore, psychological distress has been associated with gut microbiota composition, higher abundances of several bacteria belonging to the two major phyla *Proteobacteria* and *Bacteroidetes* were found in IBS patients with psychological distress, in-depth characterization of gut bacteria might lead to the discovery of new biomarkers and therapeutics [35]. It has been determined, for example, that reduction of species such as *Akkermansia muciniphila* and *Faecalibacterium prausnitzii* are present in several intestinal disorders, although little is known about the circumstances in which this may occur and about a possible link between the reduction in quantity of these species and intestinal inflammatory disorders, it is something to consider in relation to diagnosis of IBS and other GI disorders [36].

Treatment

Once IBS is diagnosed, identification of the subtype is generally used to guide treatment, the therapeutic approach in IBS is not standardized, it should be individualized and targeted on the main symptoms [37]. Elderly IBS patients’ treatment can be challenging, requiring several carefully considered approaches which, in numerous cases, render unsatisfactory results [14]. It is essential to establish a strong and empathetic relationship between the health provider and the patients in order to explore treatment options and to set realistic health goals [29], considering that the main purpose of the treatment is the reduction of symptoms and improvement of QoL; physician-patient communications is key [8]. The pharmacological treatment options are shown in Table 3; health providers should establish a predetermined period of time to test one option at a time to prove effectiveness and to adjust dosages individually [14,37].

**Table 3:** IBS pharmacological treatments First choice medications and dosages for irritable bowel syndrome (IBS) treatment (adapted from source [14]).

Irritable bowel syndrome diarrhea predominant		
-Loperamide	2-4 mg/d up to 16 mg/d	Peripheral opioid agonist
-Cholestyramine	9 g twice or thrice daily	Bile acid sequestrant
-Colestipol	2 g once or twice a day	Bile acid sequestrant
-Colesevelam	625 mg once or twice a day	Bile acid sequestrant
-Alosetron	0.5-1 mg twice daily	5HT3 receptor antagonist
-Ondansetron	4-8 mg twice daily	5HT3 receptor antagonist
-Ramosetron	5 mg once a day	Mixed opioid agonist/antagonist
-Eluxadoline	100 mg twice a day	Mixed opioid agonist/antagonist
-Rifaximin	550 mg thrice a day/14 days	Antibiotics
Irritable bowel syndrome constipation predominant		
-Psyllium	up to 30 g/day in divided doses	Soluble fiber
-Polyethylene glycol	17-34 g/d	Laxatives
-Lubiprostone	8 µg twice daily	Type 2 chloride channel activator
-Linaclotide	290 µg once daily	Guanylate cyclase C agonist
Abdominal pain		
-Dicyclomine	10-20 mg once daily	Antispasmodic
-Otilonium	40-80 mg twice or thrice/d	Antispasmodic
-Mebeverine	135 mg thrice daily	Antispasmodic
-Peppermint oil	250-750 mg twice or thrice/d	Antispasmodic
Other medications		
-Desipramine	25-100 mg/d	Tricyclic antidepressants
-Amitriptyline	10-50 mg/d	Tricyclic antidepressants
-Paroxetine	10-40 mg/d	Selective serotonin reuptake inhibitors
-Sertraline	25-100 mg/d	Selective serotonin reuptake inhibitors

Non-pharmacological Therapies

Most patients diagnosed with IBS will need pharmacotherapy [9,14]. It is known that a great deal of patients are willing to take on significant risks to mitigate the negative impact of IBS symptoms on their daily lives; health providers should be mindful of this when addressing patients in relation to the balance between risks and benefits on the different preference-based treatment approaches and the adverse effects and limitations of current pharmacotherapy [38]. IBS sufferers have increasingly sought non-pharmacological therapies in search of relief from their symptoms [8]. In this regard, the evidence-based clinical practice guidelines for IBS revised by the Japanese Society of Gastroenterology (JSGE) recommends several non-pharmacological strategies such as the low fermentable oligosaccharides, disaccharides, monosaccharides and polyols (FODMAP) diet, lifestyle and behavioral modifications, exercise included. Additionally, it is proposed the intake of probiotics, bulking polymers, and GI motility modifiers that can be prescribed regardless of the IBS subtype for better symptom management. Psychotherapy is highly recommended for IBS patients, especially those who do not respond to standard pharmacological treatment [39]. Anticipatory fear, worry, and shame regarding IBS symptoms are hallmark psychological issues in this instance. Mindfulness-based stress reduction could be useful through the cultivation of present-moment awareness which allows one to stay in the present moment with unpleasant stimuli, thereby decreasing the perceived threat [40]. Also, it has been noted that psychological stress is an important factor in the development of the syndrome; the underlying mechanism is believed to lie in the stimulation of the hypothalamic-pituitary-adrenal axis which triggers adrenocorticotropic hormone and cortisol secretion, hence influencing gut function [41]. There is evidence that tricyclic antidepressants (TCAs) and selective serotonin reuptake inhibitors (SSRIs) improve IBS symptoms [42]. On the other hand, Mind-body interventions (MBIs) such as cognitive behavioral therapy and hypnotherapy can be useful as complementary IBS treatment, interestingly yoga appears equally effective in reducing stress and improving the QoL [43]. A prospective study conducted by Lackner et al. reported moderate to substantial improvement of GI symptoms in 61% of the sample with a home-based version of cognitive behavioral therapy [44]. Other therapies target the microbiome, such as probiotics, antibiotics, and fecal microbiota transplant (FMT), as they can improve symptoms in patients with IBS related to alterations of fecal and/or gut microbiome; for instance, diarrhea-predominant IBS is associated with small intestinal bacterial overgrowth (SIBO), whereas increased levels of methanogenic Archaea, specifically *Methanobrevibacter smithii*, are associated with constipation-predominant IBS. In those scenarios, probiotics appear as a viable option [45].

Probiotics

As stated, many studies maintain that probiotics help improve IBS symptoms by stabilizing the patient's microbiota [46]. It has been argued that resetting the microbiota using antibiotics and/or probiotics could be a possible therapy for many diseases [47]; thus setting the ground for numerous clinical trials that have assessed the use or efficacy of probiotics in the treatment of GI disorders including IBS. Probiotics are being investigated and used to treat several conditions, although treatment of IBS in elderly patients is not dissimilar to that in younger patients, greater caution is needed due to the aging process or frailty in older persons, nevertheless, there is increasing scientific evidence that supplementation with probiotics seems to have positive effects on symptoms management, in particular abdominal pain and psychological distress with no adverse events reported in the sample of elderly patients studied [48]. For instance, it was reported, in a trial, the safety and usefulness of *B. longum* BB536 which improved defecation and some upper abdominal symptoms in elderly patients with chronic constipation [49]. Although it is not yet entirely understood the exact way probiotics improve GI symptoms, it is believed to be carried out by the stabilization of the intestinal microbiota [50]. Probiotics are live or attenuated microorganisms that when administered or ingested in adequate amounts confer a health benefit and may represent a therapeutic option for diseases characterized by dysbiosis such as IBS [51,52]. Various mechanisms are considered to explain the beneficial effects of probiotics in rectifying the dysbiosis of gut microbiota, including but not limited, to bacteriocin production, suppression of pathogens, enhancement of mucosal barrier function, homeostasis, immunomodulation, and neurotransmitter production [53-55]. Academic gastroenterologists and primary care physicians conducted a survey and reported that 98% of respondents considered probiotics to be safe for treating several diseases, particularly GI disorders [56]. Therapeutic gain of probiotics over placebo with a high safety profile in IBS patients has been suggested in meta-analyses, it was reported that 10 to 28 days of treatment reduced intestinal transit time; the magnitude of beneficial effects varies depending on age, presence of constipation, and bacterial strain of probiotics [57]. Diverse trial endpoints and findings are suggestive of the beneficial use of probiotics and synbiotics. For example, *Bacillus spp*. is of particular interest for its tolerance and ability to survive in environments of gastric acidity or the hostile environment of the intestine. It is thought that the positive effect of a mix of spores from five *Bacillus spp.* is due to microbiota modulation and influence on gut-brain axis communication [47]. Similarly, it has been recognized that the consumption of a two-strain mixture of *Lactobacillus* *acidophilus* over eight weeks is safe and decreases significantly flatus and composite scores compared with placebo [58]. In another trial, improvement of IBS symptom severity particularly abdominal pain was associated with *L. acidophilus* DDS-1 and *B. lactis *UABla-12 treatment [59]. Probiotics offer promising therapeutic effects, such as the oral capsule containing *Saccharomyces boulardii, B. lactis, L. acidophilus*, and *L. plantarum*, which showed considerable benefit regarding certain symptoms of patients with diarrhea-predominant and constipation-predominant IBS [60], or probiotic *B coagulans* LBSC (DSM17654) that was well tolerated, found safe, and showed significant alleviation in IBS-associated clinical symptoms like bloating/cramping, abdominal pain, diarrhea, constipation, stomach rumbling, nausea, vomiting, headache, and anxiety, compared to placebo group. *B. coagulans *LBSC treatment also improved stool consistency, decreased the severity, and conferred better QoL to IBS patients, and there was no report of adverse serious effects, which confirms that it could be used as a therapeutic supplement in the management of IBS pathophysiology and improving QoL in adults [61]. Furthermore, the findings of the analysis by Asha and Khalil establish that probiotics had the most robust effect on improving global IBS symptoms, particularly those containing multi-strains and *Bifidobacterium* species [62]. The use of the synergistic combination of prebiotics and probiotics, known as synbiotics, for IBS therapy is still in the early stages [54]; the International Scientific Association for Probiotics and Prebiotics (ISAPP) defined synbiotic as “a mixture comprising live microorganisms and substrate(s) selectively utilized by host microorganisms that confers a health benefit on the host” [55]. Thus, synbiotics refer to the combination of synergistically acting probiotics and prebiotics, which are supposed to selectively stimulate growth and/or activate the metabolism of intestinal microbiota, thus having a beneficial effect on the host’s health.

Other Preparations

Preparations that combine probiotics with other medications have shown favorable results in several studies. The addition of an antispasmodic such as simethicone to a *Pediococcus* and *Lactobacilli* formulation appears to be effective in improving IBS-related QoL scores and reducing abdominal pain and diarrhea [63]. In another placebo-controlled trial, it was found that the probiotic *B. longum* NCC3001 reduces depression scores and increases the QoL in IBS patients, these improvements were associated with changes in brain activation patterns that indicate that this probiotic reduces limbic reactivity [64]. FMT has been used for its positive therapeutic effect to alleviate the severity of IBS's myriad of symptoms, as well as depression and anxiety associated with IBS and altered microbiota. The introduction of healthy microbiota leads to restoration of gut bacterial depletion and imbalance which in turn increases diversity, enhancing *Bacteroidetes* and *Firmicutes* colonies while blocking toxic *Enterobacteriaceae*. FMT has shown great therapeutic potential for diarrhea-predominant IBS patients with anxiety and depression [65]. It is generally accepted that more clinical studies should be undertaken in large samples of diseased populations so that the assessment of their therapeutic potential provides strong evidence for their efficacy and safety in clinical use [66]. More to the point the questions of which probiotics, dosage, or for how long said probiotics or preparations should be used still remain without a unified standard answer. Quite a few international consensuses and specialist associations have concluded that probiotics reduce or alleviate IBS symptoms as well as other functional digestive disorders [55]. Extensive research is still needed to safely advocate the efficacy of the currently available therapeutic and intervention options for IBS.

## Conclusions

The elderly population is of special consideration due to aging physiological processes, geriatric syndromes, polymedicine, and multiple comorbid conditions when addressing IBS. The evidence-based information acquired through the review on the topic of IBS allows us to infer that functional GI disorders rely on biological substrates such as altered microbiota and disrupted brain-gut signaling and thus probiotics emerge as a solid option in the treatment of IBS. The overall conclusion of the analysis, given the stigmatization of patients due to the functional and organic dichotomy and the increase of older adults worldwide, is a need for consolidated guidelines such as clinical practice guides and multidisciplinary and holistic approaches to ensure comprehensive care for the aging patient population.
